# Number of casual male sexual partners and associated factors among men who have sex with men: Results from the National HIV Behavioral Surveillance system

**DOI:** 10.1186/1471-2458-11-189

**Published:** 2011-03-25

**Authors:** Eli S Rosenberg, Patrick S Sullivan, Elizabeth A DiNenno, Laura F Salazar, Travis H Sanchez

**Affiliations:** 1 Department of Epidemiology, Emory University Rollins School of Public Health, Atlanta, GA, USA; 2 Division of HIV/AIDS Prevention, National Center for HIV, Hepatitis, STD, and TB Prevention, Centers for Disease Control and Prevention, Atlanta, GA, USA; 3 Department of Behavioral Sciences and Health Education, Emory University Rollins School of Public Health, Atlanta, GA, USA; 4 Division of HIV/AIDS Prevention, National Center for HIV, Hepatitis, STD, and TB Prevention, Centers for Disease Control and Prevention, Atlanta, GA, USA

## Abstract

**Background:**

In 2006, the majority of new HIV infections were in MSM. We sought to describe numbers of casual sex partners among US MSM.

**Methods:**

Data are from the first MSM cycle of the National HIV Behavioral Surveillance system, conducted from 2003 to 2005. Relationships between number of casual male sex partners within the previous year and demographic information, self-reported HIV status, and risk behaviors were determined through regression models.

**Results:**

Among 11,191 sexually active MSM, 76% reported a casual male partner. The median casual partner number was three. Lower number of casual partners was associated with black race, Hispanic ethnicity, and having a main sex partner in the previous year. Factors associated with a higher number included gay identity, exchange sex, both injection and non-injection drug use. Being HIV-positive was associated with more partners among non-blacks only. Age differences in partner number were seen only among chat room users.

**Conclusions:**

MSM who were black, Hispanic or had a main sex partner reported fewer casual sex partners. Our results suggest specific populations of MSM who may benefit most from interventions to reduce casual partner numbers.

## Background

Men who have sex with men (MSM) have consistently been the most heavily impacted risk group in the US HIV epidemic. Recent evidence confirms that MSM in the United States [1] and in other industrialized countries [2] are experiencing a resurgence in HIV transmissions since at least 2000. In the United States, MSM of color, especially younger MSM, are particularly affected in terms of new HIV infections in recent years [3].

The reasons for racial/ethnic disparities in HIV infection are unclear, but it is likely that these disparities are explained by a complex set of behavioral, network, structural and, perhaps, biological factors [4,5]. Understanding trends in behavioral risks, and how these risks differ in subpopulations of MSM, is critical to design and disseminate HIV prevention programs to curb new HIV transmissions. A number of behavioral factors are important to HIV transmission patterns in MSM, including numbers and types of sex partners, frequency of sex, and condom use with different types of partners. Having a large number of casual male sex partners has long been recognized as an important risk factor in the transmission of HIV [6] and remains one today. A recent report from the EXPLORE study [7] found that having four or more sex partners within six months was the behavioral factor that contributed most to HIV incidence, with an attributable risk of 32.3%. Four of nine best-evidence and one of three promising-evidence interventions aimed at MSM in the 2009 Centers for Disease Control and Prevention (CDC) Compendium of Evidence-Based HIV Prevention Interventions considered reduction of partner number as an endpoint, testifying to the continued attention given to reducing partner number among MSM [8]. We used data from the first round of CDC's National HIV Behavioral Surveillance system (NHBS) [9] to describe reported numbers of casual sex partners and the factors associated with elevated partner number in a large group of MSM from 15 US cities with high HIV prevalence. Classical HIV risk factors were considered as well as each of four pre-specified interactions that, based on preliminary analyses, expert opinion, and available literature, may play an important role in understanding casual partner count. These were interactions between race/ethnicity and HIV status [10], age and HIV status [11], age and chat room usage [12], and sexual identity and having female partners [13].

## Methods

### Data source

We used data from the first MSM cycle of the National HIV Behavioral Surveillance system (NHBS-MSM1), collected from MSM in 15 metropolitan statistical areas (MSAs) from November 2003 to April 2005; participating cities have been previously reported [14]. Men were considered eligible if they were male, at least 18 years of age, current residents of participating MSAs and able to provide informed consent. Men who were determined to be eligible were invited to participate in a face-to-face interview. The NHBS-MSM1 sampling strategy and rationale have been described previously [9,15,16]. Briefly, venue-time-space sampling was used to systematically recruit participants in venues, such as bars, dance clubs, and social organizations, frequented by MSM. NHBS was classified as non-research by the CDC and was not reviewed by the CDC institutional review board (IRB); each local NHBS site reviewed the CDC study protocol and obtained approval from their respective IRBs.

### Measures

Participants were asked about the total number of men and women with whom they had sex (men: anal or oral sex; women: vaginal, anal or oral sex) in the 12 months before the interview. These total numbers of sex partners were classified, by sex of the partners, as either main sex partners ("someone you feel committed to above all others"), or casual sex partners. Exchange sex partners were considered to be a subset of casual sex partners. HIV status was determined by self-report. Other behaviors, such as use of internet chat rooms and drug use, were ascertained by self-report for the 12 months before interview.

### Statistical Methods

#### Data source

Participants eligible for inclusion in our analysis were men in NHBS-MSM1 who had at least one main or casual male sex partner within the 12 months before the interview [16]. Further restriction according to complete information on male partner number and the covariates included in our analyses resulted in the final dataset.

#### Descriptive analysis

Respondents' demographic and risk-behavior characteristics were summarized descriptively. The number of participants reporting a casual partner in the 12 months before the interview was tallied, along with the median numbers of casual and main male partners. We computed the median casual partner number separately for those who did and did not have a main male partner. The median numbers of participants' casual male partners were computed and compared across the above demographic and risk factors using Wilcoxon and Kruskal-Wallis tests.

#### Statistical modeling

To better understand the factors that were independently associated with higher casual partner count, we fit several multiple linear regression models with the number of casual partners in the 12 months before the interview as the outcome. In order to satisfy model normality and variance assumptions, a natural-logarithm transformation was applied to partner count (ln[casual partners + 1]) and participants with extreme casual partner counts were truncated at 100 [17]. Poisson regression and proportional-odds ordinal logistic regression models were also considered, but the models' goodness-of-fit assumptions were not upheld. We first fit a model that adjusted for the main effects of the following demographic factors and risk behaviors possibly associated with partner number: race/ethnicity, age, sexual identity, self-reported HIV status, education, having a main male sex partner within 12 months, having a female sex partner within 12 months, having a male exchange sex partner within 12 months (based on the construction of our outcome variable, having a male exchange partner added at least one casual male partner), MSA, chat room usage, as well as injection and non-injection drug use. We then fit interaction models that individually considered each of our four pre-specified interactions.

#### Model estimates

Least-squares means were calculated for the levels of each factor of interest by plugging the pertinent value for the factor into the estimated model, along with values for the other model terms according to their marginal distribution in the sample (observed-margins weighting). Since the casual partner counts were log-transformed, these means were then back-transformed by exponentiation and the resulting values provided estimates of the geometric mean partner count at each factor level for an 'average' person in the study sample. Since the data were found to be approximately log-normally distributed, the geometric mean partner count approximates the median count [18]. Exponentiated model coefficients estimate the geometric mean ratio (approximately the median ratio), a measure of the average relative change in casual partner number associated with each level of a factor compared to the referent group. An α level of 0.05 was used throughout for both hypothesis testing and interval estimation.

#### Model assumptions

The model fit and assumptions were evaluated. Model fit was evaluated by examining the proportion of variability explained by the model (r^2^). Model assumptions pertaining to data normality, equality of variance, and the presence of outliers were evaluated via normal probability plots, residual plots, and Cook's distance, respectively.

## Results

A total of 17,333 potentially eligible men were approached; 13,670 (79%) consented and completed an interview, and 11,471 reported sex with another man in the 12 months before the interview. We include in this report the 11,191 MSM (98% of those interviewed who reported sex with a man) who gave complete information on the number of partners and the covariates of interest.

The distributions of demographic factors and risk behaviors of interest are provided in Table 1. Forty-seven percent of MSM identified as non-Hispanic white, 18% as non-Hispanic black, and 26% as Hispanic. Most reported being HIV-negative, 13% reported being HIV-positive, and 9% had an unknown HIV status.

**Table 1:** Distribution of characteristics and model results for the number of casual male partners in the prior 12 months

Characteristic	*n*	*estimated median number * *of casual partners (95% CI)*†	*% change *‡	p-value *
**Race/ethnicity**				< .0001

White, not Hispanic	5214 (47)	3.7 (3.6, 3.9)	*ref*.	

Black, not Hispanic	2009 (18)	2.9 (2.7, 3.1)	- 23%	

Hispanic	2890 (26)	3.2 (3.0, 3.4)	- 14%	

Other §	1078 (10)	3.3 (3.0, 3.5)	- 13%	

**Age**				< .0001

18 - 24	2178 (19)	2.8 (2.7, 3.0)	- 21%	

25 - 34	3715 (33)	3.5 (3.3, 3.6)	- 4%	

34 - 44	3549 (32)	3.6 (3.5, 3.8)	*ref*.	

45 - 54	1337 (12)	3.4 (3.1, 3.7)	- 6%	

> = 55	412 (4)	3.6 (3.1, 4.1)	- 1%	

**Sexual identity**				< .0001

Homosexual	9388 (84)	3.6 (3.5, 3.7)	*ref*.	

Heterosexual	127 (1)	1.2 (0.8, 1.7)	- 66%	

Bisexual	1582 (14)	2.5 (2.3, 2.7)	- 31%	

Other	94 (1)	2.7 (2.0, 3.5)	- 26%	

**HIV status **||				< .0001

Negative	8720 (78)	3.3 (3.2, 3.4)	*ref*.	

Positive	1414 (13)	4.1 (3.8, 4.4)	+ 23%	

Untested/unknown	1057 (9)	3.0 (2.7, 3.3)	- 10%	

**Education**				0.29

Less than high school	611 (5)	3.1 (2.8, 3.5)	- 9%	

High school diploma or equivalent	1911 (17)	3.3 (3.1, 3.5)	- 2%	

More than high school	8669 (77)	3.4 (3.3, 3.5)	*ref*.	

**Main male sex partners **¶				< .0001

None	3595 (32)	6.5 (6.3, 6.8)	*ref*.	

> = 1	7596 (68)	2.4 (2.3, 2.5)	- 63%	

**Male exchange sex partners **¶				< .0001

No	10388(93)	3.1 (3.0, 3.2)	*ref*.	

> = 1	803 (7)	8.6 (7.8, 9.3)	+ 174%	

**Female sex partners**¶				0.005

None	9697 (87)	3.3 (3.2, 3.4)	*ref*.	

> = 1	1494 (13)	3.8 (3.5, 4.2)	+ 15%	

**Gone into gay or bisexual chat rooms **¶				< .0001

Didn't use	6059 (54)	2.6 (2.5, 2.7)	*ref*.	

Once a month or less	1786 (16)	3.3 (3.1, 3.5)	+ 24%	

About once a week	1044 (9)	4.2 (3.9, 4.6)	+ 61%	

Several times a week	1167 (10)	5.3 (4.9, 5.7)	+ 102%	

About once a day	744 (7)	6.3 (5.7, 6.8)	+ 138%	

Several times a day	391 (3)	6.3 (5.6, 7.1)	+ 140%	

**Injection drug use **¶				< .0001

No	10921 (98)	3.3 (3.3, 3.4)	*ref*.	

Yes	270 (2)	5.3 (4.6, 6.2)	+ 60%	

**Non-injection drug use **¶				< .0001

No	6188 (55)	2.7 (2.6, 2.8)	*ref*.	

Yes	5003 (45)	4.3 (4.2, 4.5)	+ 59%	

* p-value is for significance of the model coefficient(s)

† Model-based estimated geometric mean number of partners

‡ Calculated from the ratio of geometric mean number of partners

§ "Other" includes Asian/Pacific Islander, American Indian/Alaskan Native, multiracial men, men who specified other racial descriptions, and men who declined to report their race.

|| Self-reported

¶ In the 12 months before the interview

Among respondents, 76% reported having had a casual male partner; 32% had only male casual partners and 44% had main and casual partners; 24% had main male partners exclusively. Participants had a median of 3 casual male partners (first quartile: 1; third quartile: 9) and a median of 1 (first quartile: 0; third quartile: 1) main male partner. Those who had no main male partners during the previous year had a median of 5 casual male partners, whereas those with a main male partner had a median of 2 casual male partners (Wilcoxon p < .0001).

The results of a main-effects multivariable model of the number of casual male partners are presented in Table 1. All covariates except for education level were statistically significant (p < .0005) predictors of casual partner number, once adjusted for one another, although there was substantial heterogeneity in effect size.

The estimated adjusted median number of casual male partners was 3.7 for white MSM, 2.9 for black MSM, and 3.2 for Hispanic MSM. Therefore, in the model, compared with white MSM, black MSM has 23% fewer and Hispanic MSM had 14% fewer casual male partners. Men ages 18-24 had an estimated median of 2.8 casual male partners, whereas older men had an estimated medians of 3.4 to 3.6 casual male partners. HIV-positive participants were estimated to have had 23% more casual male partners than did negative ones. Participants who did not report a main male partner in the 12 months before the interview had an estimated 6.5 casual male partners, but those who had a main partner were estimated to have had 2.4 casual male partners. MSM who reported a male exchange-sex partner had an estimated median number of 8.6 casual male partners vs. an estimated median of 3.1 casual male partners among those with no such history. A dose-response in casual male partners was seen among chat room users, ranging from non-users, who reported an estimated median 2.6 partners, to those who used chat rooms several times a day, who reported an estimated 6.3 casual male partners. Users of either injection- or non-injection drugs had similar increases in estimated casual male partners (60% for IDU, 59% for non-injection drugs users, each compared with non-users).

**Figure 1:**
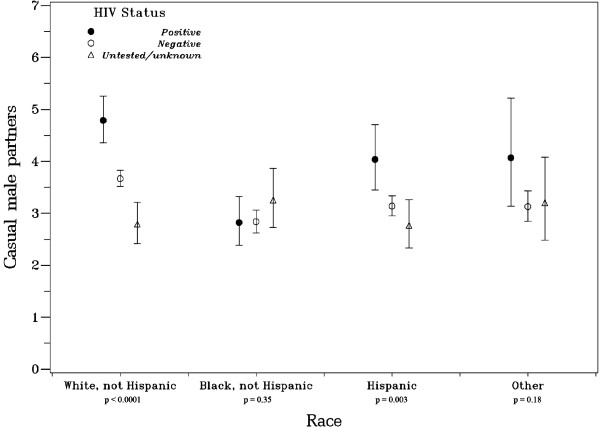
**Model-based estimated median number of casual male partners in the prior twelve months, by race/ethnicity and self-reported HIV status, among 11,191 men who have sex with men who participated in the National HIV Behavioral Surveillance System, 15 US cities, 2003-2005**.

All four interactions tested were statistically significant (p < .0001). Estimates of the median casual male partners by race/ethnicity and self-reported HIV status are shown in Figure 1. Among white MSM, significant differences in estimated casual male partners by HIV status were seen: positive men reported an estimated 4.8 casual male partners whereas negative men reported an estimated 3.7 casual male partners. In contrast, the median number of 2.8 casual male partners was not significantly different between HIV-positive and -negative black MSM. Among Hispanic MSM there was a more modest but significant difference in casual partner number, compared to white MSM: HIV-positive men had an estimated 4.0 casual male partners while HIV-negative men had 3.1.

**Figure 2:**
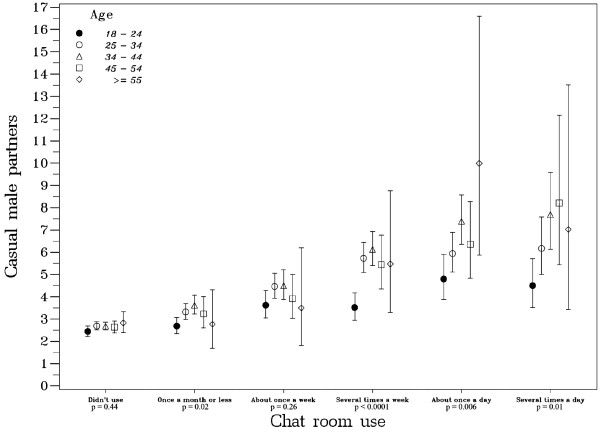
**Model-based estimated median number of casual male partners in the prior twelve months, by age and chat room usage, among 11,191 men who have sex with men who participated in the National HIV Behavioral Surveillance System, 15 US cities, 2003-2005**.

Figure 2 displays the associations of age group and the number of casual male partners by chat room usage. Among those who did not use chat rooms, the number of casual male partners was relatively constant across age groups, with participants in the youngest age group indicating an estimated median of 2.4 casual male partners and those ages 45 to 54 reporting an estimated median of 2.6 casual male partners. Yet the number of casual male partners increased with the frequency of chat room usage, and differentially so by age. For example, among MSM who used chat rooms several time a week, those between 18 and 24 years old had an estimated median of 3.5 casual male partners, whereas MSM ages 45 to 54 had an estimated median of 5.4 casual male partners.

Among those who used chat rooms most frequently (several times a day), the difference was even greater; those between ages 18 and 24 had an estimated median of 4.5 casual male partners, whereas MSM ages 45 to 54 had an estimated median of 8.2. Forty-six percent of men 18 - 24 years of age reported no use, while 62% of those aged 45 - 54 years reported no use. Twenty-two percent of MSM aged 18 - 24 years and 17% of those between 45 and 54 years used chat rooms several times a week or more (a level of use at which highly significant differences in casual partner count are seen by age among MSM).

An examination of the interaction between HIV status and age group revealed heterogeneity in the association between being infected with the virus and an increase in the number of casual male partners. Among MSM ages 18 to 24 years, HIV-positive men had an estimated median of 2.8 casual male partners (95% CI: 1.8, 4.1) and negative men had an estimated median of 2.9 partners (95% CI: 2.7, 3.1). Yet among MSM ages 25 to 34 years, HIV-infected MSM had more casual male partners on average; HIV-positive MSM had an estimated median of 5.0 partners (95% CI: 4.4, 5.8) and HIV-negative MSM had an estimated median of 3.3 (95% CI: 3.1, 3.5). Among MSM aged ≥ 55 years, no such difference was evident; HIV-positive men had an estimated median of 3.8 (95% CI: 2.6, 5.4) casual male partners while HIV-negative men also had an estimated median of 3.8 (95% CI: 3.2, 4.4).

Modeling of the interaction between sexual identity and reporting a female sex partner in the 12 months before the interview provided further insight. Having a female partner was significantly associated with higher numbers of casual partners only when the respondent identified as homosexual (p < .0001) but did not significantly change estimates among those identifying as heterosexual, bisexual, or 'other'. Homosexual men who had a female sex partner had an estimated median of 4.7 (95% CI: 4.1, 5.3) casual male partners, whereas those who had no female partner had an estimated median of 3.5 [95% CI: 3.4, 3.6).

Model fits were good; the main-effects model had an r^2 ^of 0.26, indicating that 26% of casual male partner count variability was explained by the covariates modeled. Examinations of normal probability and residual plots, as well as of Cook's distance indicated that model assumptions were upheld.

## Discussion

MSM of color reported fewer casual male partners than did their white counterparts, providing further evidence that partner number is not driving the long-observed disparity in HIV incidence between racial/ethnic minority men and white men. A recent meta-analysis of risk differences in black and white MSM reported a 36% decrease in the odds of having more partners (main and casual) among black MSM, compared to white MSM, using data from 10 studies [5]. Our data provide additional information about this factor for Hispanic MSM, for whom there has been less focus on individual risk behaviors relative to white MSM.

Furthermore, although white and Hispanic self-reported HIV-positive MSM indicated having more casual male partners than did their HIV-negative counterparts, equivalent numbers of partners were seen among black MSM across HIV-status. Several possible explanations for this may exist. One is that black MSM tend to be less aware of their true serostatus, and thus self-reported HIV status may appear to have a weaker relationship with casual partner number because of misclassification. A five-city sub-study of these NHBS-MSM1 participants that performed HIV rapid testing, with laboratory confirmation, found that 67% of seropositive black MSM were unaware of their status, compared to 18% of seropositive white MSM [19]. This higher misclassification of self-reported HIV status may 'smooth out' any differences in casual partner number reported among black MSM. Alternatively, transmission among black MSM may be more related to other risk-behaviors or mechanisms than to having more casual partners, compared to white and Hispanic MSM.

Although young men ages 18 to 24 years tended to have fewer casual male partners overall, we found that it was important to interpret this observation in the context of chat room use. There has been a growing interest in the association between using the internet to meet MSM partners and the practice of higher-risk sexual behavior [20,21]. While the direction of causality is still unclear, Mustanski has reported in a prospective diary study of 113 MSM that it was those who practiced riskier sex (UAI) who tended to find their partners online, rather than the reverse [22].

As frequency of chat room use increased, so did the reported number of partners, with greater increases reported by older MSM. While the magnitude of partner counts among frequent users is striking, it is important to be aware of how many respondents had such levels of usage. About a fifth of MSM used chat rooms several times a week or more, a level of use at which a highly significant difference in casual partner count was seen between younger and older MSM. Thus we see that age disparities in casual partner count among MSM are concentrated in a minority that uses gay or bisexual chat rooms heavily.

There are several possible hypotheses for the observed differences of chat room usage by age. It may be that younger men use MSM chat rooms more for general socialization, such that high usage is less associated with a propensity to find partners, compared to older MSM who may use chat rooms more exclusively for meeting partners. Alternatively, there may be a generational difference in nomenclature, where younger MSM associate the term "chat rooms" with a different array of services (such as social networking websites) than do older men. Whatever the reasons underlying this age difference, it may be more important to target interventions towards older MSM who frequently use chat rooms.

Having had a male exchange sex partner was the factor associated with the largest increase in casual male partners. Exchange sex has long known to be a correlate of HIV risk behaviors [23,24] and specifically of an increased numbers of casual partners [25,26].

We observed, as have others [27], that MSM reporting a main male partner within the previous year had on average substantially fewer casual male partners. While having a main partner appears to exert a 'protective' effect on the level of casual partners, this does not necessarily equate with a reduction in HIV transmission risk. A separate analysis among a subset of these NHBS-MSM1 participants estimated that a majority of HIV transmission among MSM was attributable to main partners [28].

Both injection and non-injection drug use were associated with similar increases in casual male partners, but non-injection drug use was far more prevalent. A previous study of methamphetamine use among gay and bisexual men found 97% and 63% increases in the mean number of sex partners in the previous 12 months, respectively [29], while others have helped to establish the link between a variety of non-injection drugs with risky sexual practices in MSM [30]. In another cohort of MSM, the unadjusted hazard ratios for seroconversion among users of different non-injection drugs were similar to or slightly larger than that for injection drug users [7].

There are some important limitations of our analysis. First, although NHBS-MSM1 used a sampling methodology designed to get a minimally-biased sample of venue-attending MSM in the cities surveyed, our respondents are not representative of all MSM in the United States or in participating cities. Some responses may have been affected by recall and social desirability biases. Caution should also be used when interpreting the estimated median partner counts literally. Each estimate reflects an 'average' person who possesses the other modeled characteristics in proportion to their frequencies in the sample. This method of estimation is superior to weighting the levels of the characteristics equally, but it is essential to bear in mind that these estimates reflect no true group or person and the relative differences within factors are most telling.

There were also limitations in our data about chat room usage. Our questionnaire asked about chat room usage only, rather than the broader array of social networking services currently in use. The ways in which MSM use the internet have proliferated and diversified since the time the survey was designed during the early 2000 s. Although we did not measure the usage of web services overall to meet partners, we appear to have captured an online effect to some extent, one that might even be larger had we measured other online services such as social networking sites.

Having a large number of casual male sex partners is an established risk-factor in the transmission of HIV, and higher casual partner number increases one's chances of encountering an HIV-discordant partner. An earlier analysis of NHBS-MSM1 data documented that 24% and 25% respondents from NHBS-MSM1 reported unprotected insertive and receptive anal sex with their last casual partners, respectively, underscoring that the HIV acquisition risk associated with high partner number remains real [16]. Furthermore, higher partner numbers may be indicative of increased sexual concurrency, which has been demonstrated to amplify HIV transmission potential [31].

## Conclusions

The reemerging MSM epidemic in the United States is comprised of multiple smaller epidemics in subgroups of MSM. HIV prevention programs for MSM should be developed and prioritized based upon a deep understanding of behavioral risks within different subgroups. Based on our findings, prevention programs focusing on reducing numbers of casual sex partners in the United States should focus on white, non-Hispanic men; homosexually identified men; men engaged in exchange sex; men with female partners; and men with recent non-injection drug use. The association of chat room use and higher casual sex partner numbers, especially for older men, suggests that prevention programs targeting reduction in numbers of casual sex partners should be considered for use in chat rooms settings and other online venues where MSM congregate. Our data also reinforce the understanding that racial differences in the numbers of casual partners do not explain the disparity in HIV prevalence in black MSM in the United States.

## Competing interests

The authors declare that they have no competing interests.

## Authors' contributions

ESR led statistical analysis and manuscript drafting. PSS participated in statistical analyses, and manuscript preparation. EAD oversaw collection of the NHBS data, provided technical assistance for data analyses, and assisted in manuscript preparation. LFS led data collection the Atlanta site, collaborated in data analyses, and assisted in manuscript preparation. THS provided scientific oversight for collection of the NHBS data, collaborated in the conception of the analytic plan, and assisted in manuscript preparation. All authors read and approved the final manuscript.

## Pre-publication history

The pre-publication history for this paper can be accessed here:

http://www.biomedcentral.com/1471-2458/11/189/prepub
